# Apoptotic Pathways Linked to Endocrine System as Potential Therapeutic Targets for Benign Prostatic Hyperplasia

**DOI:** 10.3390/ijms17081311

**Published:** 2016-08-11

**Authors:** Letteria Minutoli, Mariagrazia Rinaldi, Herbert Marini, Natasha Irrera, Giovanni Crea, Cesare Lorenzini, Domenico Puzzolo, Andrea Valenti, Antonina Pisani, Elena B. Adamo, Domenica Altavilla, Francesco Squadrito, Antonio Micali

**Affiliations:** 1 Department of Clinical and Experimental Medicine, University of Messina, Azienda Ospedaliera Universitaria Policlinico “G. Martino”, 98125 Messina, Italy; mrinaldi@unime.it (M.R.); hrmarini@unime.it (H.M.); nirrera@unime.it (N.I.); andrea.valenti@unime.it (A.V.); fsquadrito@unime.it (F.S.); 2 Department of Human Pathology, University of Messina, Azienda Ospedaliera Universitaria Policlinico “G. Martino”, 98125 Messina, Italy; giovanni.crea@unime.it (G.C.); cesare.lorenzini@unime.it (C.L.); 3 Department of Biomedical and Dental Sciences and Morphofunctional Imaging, University of Messina, 98125 Messina, Italy; puzzolo@unime.it (D.P.); apisani@unime.it (A.P.); elenabianca.adamo@unime.it (E.B.A.); daltavilla@unime.it (D.A.); amicali@unime.it (A.M.)

**Keywords:** benign prostatic hyperplasia, apoptosis, treatment

## Abstract

Benign prostatic hyperplasia (BPH) is a chronic condition common in older men that can result in bothersome lower urinary tract symptoms. The molecular mechanisms and networks underlying the development and the progression of the disease are still far from being fully understood. BPH results from smooth muscle cell and epithelial cell proliferation, primarily within the transition zone of the prostate. Apoptosis and inflammation play important roles in the control of cell growth and in the maintenance of tissue homeostasis. Disturbances in molecular mechanisms of apoptosis machinery have been linked to BPH. Increased levels of the glycoprotein Dickkopf-related protein 3 in BPH cause an inhibition of the apoptosis machinery through a reduction in B cell lymphoma (Bcl)-2 associated X protein (Bax) expression. Inhibitors of apoptosis proteins influence cell death by direct inhibition of caspases and modulation of the transcription factor nuclear factor-κB. Current pharmacotherapy targets either the static component of BPH, including finasteride and dutasteride, or the dynamic component of BPH, including α-adrenoceptor antagonists such as tamsulosin and alfuzosin. Both these classes of drugs significantly interfere with the apoptosis machinery. Furthermore, phytotherapic supplements and new drugs may also modulate several molecular steps of apoptosis.

## 1. Introduction

The most common non-malignant urological disease among aging men is the benign prostatic hyperplasia (BPH), which affects more than 40% of individuals over the age of 60 [1]. It is a progressive disease, which causes an increase in prostate volume, a decrease in maximum urinary flow rate, as well as the development of acute urinary retention (AUR) [2]; thus, it represents a risk in terms of health [3]. In the next few years, the rate of male population treated for BPH will increase as an outcome of aging [4,5].

In adult men, BPH is commonly characterized by lower urinary tract symptoms (LUTS) in association with sexual dysfunctions (SD), such as erectile and ejaculatory dysfunction, decreased libido, and overall dissatisfaction [3]. Furthermore, LUTS is often associated with diabetes mellitus, urinary tract infections, and neurological disorders [3]. Preclinical and clinical studies showed that LUTS and SD share similar pathogenic mechanisms [6].

The specific processes leading to the phenotype observed in BPH have not yet been completely clarified. A lot of evidence indicates a molecular link between androgens, estrogens, growth factors, and/or neurotransmitters in the evolution of BPH. The increase of prostate volume is caused by a complex and gradual growth involving both prostate glandular epithelium and fibromuscular stroma [7], primarily in the transitional zone [8].

Cell growth in BPH may also cause nodules in the periurethral region of the prostate, able to partially or completely obstruct the urethra [9]. Recent evidences showed a series of pathological conditions in BPH, such as chronic inflammation [10], deregulation of circulating hormonal levels, and abnormal tissue remodeling [11,12]. These processes also include an altered expression of cytokines and chemokines [13], a disturbance of immune surveillance and recognition, as well as a pathologic modification of stroma, observed in several fibroplasias and in different malignant tissues [7,12,14]. The development of prostate is linked to the endocrine system; in particular, epithelium and mesenchyme are controlled by androgens. For these reasons, many of the diseases affecting the prostate are correlated to the endocrine system and their treatments are directed at the manipulation of this very complex system [15].

While it is known that in the genesis of prostate cancer an unbalance between cellular proliferation and cell death plays a prominent role, no concordant data are currently available in the genesis of BPH [16,17]. Apoptosis, or programmed cell death, is a complex process implicated in development and cellular stability [18].

Apoptosis is primarily triggered by specific stimuli, such as an activation or inactivation of several molecules through a multifaceted regulation [19]. Apoptosis is also involved in embryonic development and homeostatic maintenance of tissues and organs [20,21], being regulated by different death- or survival-related genes [22]. Programmed cell death includes many molecular steps that culminate in the clearance of impaired and altered cells, while at the same time avoiding the leaking of deleterious substances into the surrounding tissues [23,24].

Overall, the identification of the apoptotic pathways involving the prostatic cells undergoing hyperplasia may offer new data on the carcinogenesis, thus providing novel therapeutic targets [17].

## 2. Endocrine Control of Prostatic Growth

In BPH, an important role is played by hormonal imbalance; in fact, the development and the growth of the prostate is controlled by a synergy of normal levels of androgens and estrogens [25] and, consequently, by a controlled balance between cell growth and apoptosis [16].

**Figure 1:**
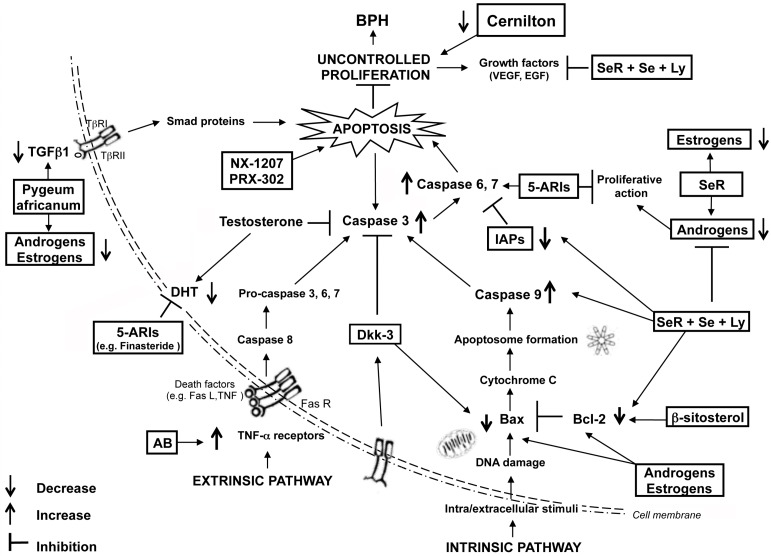
Schematic representation of the endocrine-linked apoptotic mechanisms involved in benign prostatic hyperplasia (BPH) and of its medical treatments.

In fact, dihydrotestosterone (DHT), which binds the androgen receptor (AR), strategically modulates the proliferation and increase of the prostate volume [26]. An adequate level of androgens is necessary to regulate the growth of prostate [27]: in fact, androgen deficiency leads to a reduction of the prostatic glandular epithelium [28,29]. Interestingly, stromal cells can use the signaling pathways of growth factors—such as epidermal growth factor (EGF), basic fibroblast growth factor (bFGF), insulin-like growth factor (IGF), and transforming growth factor β1 (TGFβ1) [30] (Figure 1)—to activate genes sensitive to androgens, although in an androgen-independent way [31]. These paracrine pathways are important in the regulation of proliferation and apoptosis of prostate epithelial cells [32].

Androgens are also implicated in the development of BPH and prostate cancer. In a model of castrated rats, testosterone inhibits cell death in the ventral prostate by controlling procaspase and caspase-3 and -6 mRNA levels, as well as active proteins levels [33]. In BPH, the abnormal growth is related to the activation of proliferative processes, and vice versa for the inhibition of apoptotic pathways, which is induced by androgen stimulation, thus demonstrating a key role of DHT in the development of the disease [34].

Another important role in BPH development is played by estrogens (Figure 1), even if concordant data are not present. In fact, serum estrogens were reported to be correlated with prostate volume and other aspects of BPH [35]. On the contrary, this relationship was not found by Miwa et al. [36]. The types of estrogens and of estrogen receptors (ER) may influence the estrogen action in the prostate; similarly, stromal cells from BPH may respond differently from normal stromal cells to estrogenic ligands.

Indeed, the true roles of ER-α and ER-β in BPH have not yet been elucidated. In particular, ER-α activation causes hyperplasia, inflammation, and dysplasia of the prostatic tissue [37]. On the contrary, ER-β reduces cellular proliferation in the prostate and activates apoptosis in BPH in an androgen-independent manner [38]. In fact, in ER-β knockout (ER-β KO) mice, prostatic hyperplasia progresses with age, which differs from wild-type or ER-α KO mice [39]. In this way, the ratio ER-α/ER-β may play an important role in estrogen-induced proliferation.

## 3. Molecular Pathways of the Apoptosis Machinery and Benign Prostatic Hyperplasia (BPH)

It is well known that normal cells grow through a delicate equilibrium between the stimulation and the inhibition of specific pathways of cellular death. Among them, an important role is played by the genes encoding B cell lymphoma (Bcl)-2 and Bcl-2 associated X protein (Bax) [40]. In particular, cells showing a higher expression of Bax undergo apoptosis, while those overexpressing Bcl-2 often undergo carcinogenesis, a condition characterized by a suppression of apoptosis [41].

In the prostate, the expression of anti-apoptotic protein Bcl-2 is limited to basal epithelial cells, which are able to resist androgen deprivation [42]. In BPH, the expression of Bcl-2 in epithelial cells is low, while the activity of Bax is high; in particular, Bax activity is positively linked with age [43].

The upregulation of Bcl-2 was shown to be related to the prognosis of prostate cancer advance in hormone-deprivation, augmented tumor stage, and poor outcome [42,44]. It has been suggested that the Bax/Bcl-2 ratio is very important for the androgen regulation of apoptosis, determining cell fate; indeed, an increase in this ratio during apoptosis, induced by androgen withdrawal, has been observed [12,45] (Figure 1).

The apoptotic machinery of prostate cells is modulated by TGFβ, which stimulates cell death and involves several transcription factors [46,47,48,49].

In particular, TGFβ1 regulates extracellular matrix production and degradation, cell differentiation, and proliferation [50,51]. This cytokine plays a role in regulating prostate growth [11] and induces apoptosis in prostate epithelial cells [52]; in response to terazosin or castration, an upregulation of TGFβ1 expression determines prostatic epithelial apoptosis [53]. In BPH, TGFβ shows an inhibitory role, as it controls proliferation and stimulates apoptosis in epithelial cells [54].

In prostatic epithelial cells, proliferative activity is under control of different signaling pathways, among which the glycoprotein Dickkopf-related protein 3 (Dkk-3) is included [55]. This glycoprotein is encoded by a gene at the 11p15 locus, usually eliminated in tumors [56], and includes four proteins named Dkk-1, Dkk-2, Dkk-3 and Dkk-4 [57]. Dkk-3 does not modulate Wnt signaling proteins, a family of paracrine signaling growth hormones that plays a crucial role especially in development and carcinogenesis [56,58,59,60]. An in vitro study on the effects of Dkk-3 on prostatic cell growth revealed that cellular action of Dkk-3 is mediated by receptors on the cell surface [59]. However, other studies reported actions against proliferation, or vice versa, upon Dkk-3 overexpression, likely induced by the endoplasmic reticulum stress [59].

Dkk-3 is mainly expressed in the epithelial tissue in physiological conditions, while in the prostatic disease it is elevated in the stroma, predominantly in endothelial cells [59]. Moreover, an overexpression of this glycoprotein in BPH causes an inhibition of the apoptosis machinery through a reduction in Bax expression [59]. Again, a high rate of Dkk-3 in vessels downregulates local expression of angiopoietin-1, that, in turn, leads to vessel destabilization and sprouting of microvessels into the stroma [56]. These data further confirm the pathophysiological implications of stromal remodeling in BPH [47,49]. Indeed, the typical molecular and biological functions of Dkk-3 are still not clear, and also the role of high levels of Dkk-3 in the prostatic stroma disease is not clearly identified.

Apoptosis in BPH is also regulated by the inhibitors of apoptosis proteins (IAPs), able to interfere with caspases [24]. To date, eight mammalian IAPs are known: X-chromosome-linked IAP (XIAP) [61], cellular IAP-1 and -2 (cIAP-1 and cIAP-2), neuronal apoptosis inhibitory protein (NAIP), survivin [62], BRUCE, livin- and testis-specific IAP (Ts-IAP). In testis, IAP-like protein 2 (ILP-2), a tissue-specific homologue of XIAP, directly inhibits caspase-9. Increased IAPs expression has been shown in pathological human prostate including BPH, prostatic intraepithelial neoplasia, and cancer [63]. cIAP-1 and cIAP-2 were demonstrated on the basis of their capability to bind tumor necrosis factor-associated factor 2 (TRAF2) and they are mainly involved in the regulation of the extrinsic pathway of the apoptosis through the modulation of caspases activity [64]. Another member of IAPs family, survivin, is highly expressed in embryonic tissues and is also present in prostate cancer [62,65].

In experimental BPH, cIAP-1, cIAP-2, NAIP, and survivin have been detected by molecular analysis [24], so that IAPs might be confirmed as diagnostic markers in different pathologies [66] (Figure 1).

## 4. Treatments of BPH

The clinical approach to the treatment of BPH has changed considerably over the past 20 years, and medical treatment is nowadays preferred [67,68,69,70]; in fact, the number of prostatectomies for BPH-related diseases in the United States has progressively lowered [71]. In a recent study [5], it was shown that the majority of BPH patients (54.8%) are managed with drugs, while only 1.1% undergoes surgical procedures.

The reduction of surgical treatment can be also related to better-tolerated and effective medical treatments [72], such as the αl-adrenergic-receptor antagonists (α1-ARAs) and/or 5α-reductase inhibitors (5-ARIs) [70].

Recently, particular attention was focused on the healthcare and management of BPH and relative complications, such as AUR and others [5]. Classical drug targets control the increase in the prostatic size (static component) or in the tone of smooth muscle cells (dynamic component) in BPH [70]. The better treatments in terms of efficacy for BPH are considered those reducing the tone of smooth muscle cells [69]. Finasteride and dutasteride act by inhibiting the proliferative action of androgens; conversely, α1-ARAs, such as tamsulosin and alfuzosin, target the dynamic component of BPH [70]. Both these drug classes significantly interfere with the apoptosis machinery. The main targets of current medical therapies for LUTS/BPH are to (i) ameliorate bothering symptoms; (ii) improve life quality; and (iii) prevent disease progression.

Conventional medical treatments of symptomatic BPH include: (i) α1-ARAs; (ii) 5-ARIs; and (iii) the combination of α1-ARAs and 5-ARIs. α-Blockers (αB) or α1-ARAs, 5-ARI, anticholinergics, and their associations are usually employed in the treatment of male LUTS [73,74,75,76]. However, some drugs for the treatment of LUTS/BPH may cause sexual dysfunction, with interclass and intraclass drug effects differences [77,78].

The growing interest in phytotherapy made available new therapeutic alternatives for various medical conditions, including BPH. Along with αB and 5-ARIs, *Serenoa repens* (SeR) is without doubt the most widely used phytotherapic. Together with Pygeum africanum, SeR is available in many European countries for symptomatic BPH [77].

Phytotherapy for the treatment of LUTS in association with BPH is common also in most of western countries. In Germany and Austria, phytotherapy represents more than 90% of all treatments prescribed for BPH, and its use has increased considerably in the USA [77,79]. Epidemiological studies showed that several patients have chosen a nonsurgical therapy for BPH, such as a phytotherapic approach alone or in association with other drugs [79,80]. Consequently, in the last years, many efforts to assess the clinical evidence on these alternative treatments for BPH have been conducted [81].

Finally, recent evidences pointed out the positive role of NX-1207, a therapeutic protein with selective pro-apoptotic properties, in BPH therapeutic management [82].

## 5. α1-Blockers

The α1-ARAs, including alfuzosin, doxazosin, tamsulosin, and terazosin, are considered (from the American Urological Association Guidelines in 2010) the most common therapy for BPH-related LUTS [72]; all of these drugs are equally efficacious, even if they present adverse effects [72].

The α1-ARAs’ mechanism of action in BPH is the blockade of α1-adrenergic-receptors (α1-ARs), which are particularly present in the smooth muscle cells of the prostate and of the bladder neck [83].

To date, three α1-AR subtypes, α_1A_, α_1B_ and α_1D_, have been identified. The α_1A_ subtype is usually implicated in the regulation of the tone of smooth muscle cells in the prostate and in the bladder neck, while the α_1B_ subtype modulates blood pressure by contracting the smooth muscle cells in the blood vessels [83]. The α_1D_ subtype is probably involved in the contraction of the bladder muscle and in innervations of sacral spinal cord [83]. Acting on these receptors, α1-ARAs relax prostatic smooth muscle cells and improve urinary flow, as well as LUTS and BPH-related bladder outlet obstruction [84].

Furthermore, it was shown that α1-blocker doxazosin triggers prostate cell apoptosis in BPH patients [85]. Doxazosin and terazosin block α1-adrenergic innervations and relax smooth muscle cells in the prostate; however, this action only partially accounts for the long-term clinical effects in the treatment of BPH [86,87].

Experimental and clinical studies were performed to elucidate whether the activation of apoptosis in prostate cells by α1-adrenoceptor antagonists could represent a key molecular mechanism justifying their long-term efficacy in the management of BPH-associated LUTS and in the potential reduction of prostate cancer growth [88].

In this context, it has been suggested that apoptosis represents a good target for the long-term therapeutic impact of doxazosin and terazosin in BPH [89]. Different studies demonstrated that doxazosin could induce apoptosis in benign and malignant cells of prostate through a mechanism mediated by tumor necrosis factor receptors (TNFRs) [12,89]. Interestingly, TNFRs’ self-assembly process should be recognized as one of the potential mechanisms of triggering apoptosis [90].

Moreover, the apoptotic effect of doxazosin and terazosin, elicited without involving cell proliferation in prostate cancer, may have high clinical significance in the management of the disease [86]. This effect is confirmed by the presence of different mechanisms, independent from α1-adrenoceptor; in fact, tamsulosin, a sulfonamide-based α1-antagonist, was not able to induce an apoptotic response [91].

Many randomized clinical trials indicated the efficacy of various α1-ARAs in the treatment of BPH. Furthermore, α1-ARAs are characterized by a rapid onset to action, a high urine flow rate, and a significant improvement in patients’ symptom scores. In addition, α1-ARAs show a good profile of safety, thus representing a valuable choice of first-line treatment in patients with moderate to severe LUTS [92,93,94,95].

Overall, the significant relationship between apoptosis activation and symptom scores of BPH amelioration in patients with prostate cancer suggests that enhanced apoptosis is a possible therapeutic goal, also considering the long-term efficacy of doxazosin in the LUTS treatment [86]. It must be kept in mind that the abovementioned effect is not only typical of doxazosin: in fact, terazosin treatment induced apoptosis in prostate cells of BPH patients, with no effect on the cellular proliferation [85]. In addition, an experimental model of BPH documented the doxazosin capability to cause prostate cell death without affecting their proliferative capacity [96].

In vitro studies demonstrated that the apoptotic action is exclusively achieved by the quinazoline-based α-adrenoceptor antagonists. Again, malignant prostatic epithelial cells, in association with prostatic benign epithelial and smooth muscle cells, activate apoptosis after treatment with quinazolines in a dose-dependent manner [97] (Figure 1).

## 6. Finasteride

Finasteride is a selective 5-ARIs type II isoenzyme that prevents the conversion of testosterone to DHT in the prostate, causing a reduction of the gland size via induction of apoptosis [31].

Inhibitors of 5α-reductase reduce the size of BPH tissues through the activation of apoptosis, but their mode of action is still not clear. These drugs relieve symptoms of bladder outlet obstruction and reduce the risk of AUR [98]. Both α1-blockers and 5-ARIs cause apoptosis in the prostate gland, without affecting cellular proliferation [86,99].

These data correlate well with the concept that inhibition of DHT production in the prostate triggers apoptosis without affecting DHT-stimulated cellular proliferation [100] (Figure 1), and in agreement with the evidence that patients with 5α-reductase deficiencies never develop prostate cancer [53,101]. Five milligrams daily of finasteride for four years produced a significant reduction in serum levels of DHT [102,103].

Interestingly, finasteride therapy prevents AUR and delays the need for invasive therapy in responders. Finasteride shows good efficacy, particularly in men with larger prostates [104], also reducing typical symptoms associated with BPH, like hematuria [105,106,107,108]. An increase in cell death by apoptosis and a reduction of microvessel density in BPH human samples were demonstrated after finasteride treatment [31].

Finally, finasteride administration induced the apoptosis cascade in BPH tissues by activating effector caspases-3 and -6; this effect was transient, because the apoptotic process was no longer observed after about one month of treatment [98] (Figure 1).

## 7. Phytotherapic Supplements

Phytotherapic compounds such as β-sitosterol, Pygeum africanum, Cernilton, and SeR showed good efficacy on the symptoms and the urinary flow measures related to BPH, with mild and infrequent adverse events [79,81,109,110,111,112,113,114].

β-Sitosterol, a phytosterol mainly originating from South African star grass, acts by inhibiting 5α-reductase, the predominant enzyme in the prostatic metabolism of testosterone. It improves urological symptom scores and urodynamic measurements, including maximum flow rate and post-void residual urine volume (PVR); moreover, this phytotherapic compound shows mild adverse effects, without a significant difference in adverse event rates compared with placebo [110,111,114].

An extract of the African evergreen tree Pygeum africanum may disable androgen receptors by blocking their nuclear translocation and inhibiting cellular growth factors, such as fibroblast and epidermal growth factors [80] and TGFβ1 [115]; in addition, it has also an anti-estrogen and anti-inflammatory effect [26]. Pygeum significantly ameliorates BPH symptoms, such as nocturia and PVR, and shows minor adverse events, such as rare gastrointestinal problems [112].

Cernilton, an extract from ryegrass pollen, protects acinar epithelial cells, inhibits stromal proliferation, and acts on smooth muscle tone; furthermore, it enhances apoptosis and shows antiandrogenic effects [116]. It improves BPH subjective symptoms, including nocturia, without significant difference in urodynamic measures when compared with placebo [109,116,117].

SeR, an extract from the berry of the American saw palmetto or dwarf palm plant [118,119,120,121], induces evident LUTS relief. Its proposed mechanisms of action are the following: (i) antiestrogenic and antiandrogenic effects [122] by weakly inhibiting the conversion of T to DHT [123]; (ii) modulation of apoptosis [78,124]; (iii) inhibition of 5-ARIs in the stroma and epithelium of the prostate [125]; and (iv) relaxation of smooth muscle cells of the detrusor through interaction with α1-adrenergic receptors [126]. SeR is prescribed alone or in association with other natural substances, such as selenium (Se) and lycopene (Ly). These compounds were recently investigated in an experimental model of testosterone-dependent BPH [24] and in clinical trials [127].

It was demonstrated that BPH animals had enhanced expression of NAIP and survivin, whereas the association of SeR, Se, and Ly exerted the highest activity in inhibiting IAPs, stimulating apoptosis, and reducing prostate enlargement [24]. Also growth factors, such as vascular endothelial growth factor (VEGF) and epidermal growth factor (EGF), were inhibited by treatment with Se-Ly-SeR combination [113,120] (Figure 1).

## 8. NX-1207

NX-1207 is a novel cysteine-containing linear protein [128] and is also the first drug in phase III trials suitable for BPH patients [129]. It is administered to BPH patients through a transrectal intraprostatic inoculation under ultrasound guidance [130]. Different studies have shown that NX-1207 activates apoptosis [82,130,131,132,133,134] in BPH tissue, causing a reduction in the prostate volume with symptomatic improvement [130]. In in vitro studies, NX-1207 positively controlled apoptotic markers such as caspases and annexin V [82].

Clinical evidence revealed that NX-1207 caused a substantial symptomatic improvement and, if compared with AB and 5-ARIs, did not show any kind of compliance issues even in the elderly people undergoing multiple therapies. Finally, NX-1207 has not revealed any kind of sexual side effects (impotence, loss of the libido, etc.) [132] (Figure 1).

## 9. PRX-302

PRX-302, known also as topsalysin, is a modified recombinant protein able to be selectively activated by prostate specific antigen (PSA), causing localized cell death and tissue disruption without any damage of the neighboring tissues [128]. PRX-302 binds to glycosylphosphatidyl inositol (GPI) receptors placed on the cell surface of prostate cells. Once activated by PSA, PRX-302 combines with other similar molecules, forming a stable transmembrane pore that activates cell death [135]. PRX-302 is currently being tested in the treatment of LUTS in BPH.

Clinical evidence revealed that the intraprostatic injection of PRX-302 significantly reduced the International Prostate Symptom Score (IPSS), with mild to moderate transient adverse events [136]. The treatment did not show any negative effects on the erectile function [129] (Figure 1).

## 10. Conclusions

LUTS are commonly observed in patients with BPH, which is considered an increasing problem for public health. Even if some successes in treating BPH patients with α-adrenoblockers and 5-ARIs have been achieved, the combined use of the drugs is appropriate because the differences in mechanisms of action permit both to act on the smooth muscle tissue, producing its relaxation, and to reduce the size of prostate by the induction of apoptosis, which ultimately induces the maximum therapeutic effect [137].

An uncontrolled growth of both the glandular and the connective tissue cells in the prostatic transitional zone is involved in the development of BPH. While in the pathogenesis of prostatic tumor an imbalance between cellular proliferation and cell death plays a prominent role, no concordant data are currently available about its role in the genesis of BPH. However, hormonal imbalance plays an important role in BPH; in fact, the normal development of the prostate is obtained through a balance between cell growth and apoptosis, which are regulated by normal levels of androgens and estrogens. Therefore, the development of new therapeutic approaches for BPH requires the knowledge of the molecular pathways involved both in the proliferation and in the programmed death of prostate cells.

## Abbreviations

BPH, Benign prostatic hyperplasia; AUR, Acute urinary retention; LUTS, Lower urinary tract symptoms; SD, Sexual dysfunctions; DHT, Dihydrotestosterone; AR, Androgen receptor; EGF, Epidermal growth factor; bFGF, Basic fibroblast growth factor; IGF, Insulin-like growth factor; TGFβ1, Transforming growth factor β1; Bcl-2, B cell lymphoma gene-2; Dkk-3, Dickkopf-related protein 3; PI3K, Phosphoinositide 3-kinase; IAPs, Inhibitors of apoptosis proteins; XIAP, X-chromosome-linked IAP; cIAP-1 and cIAP-2, Cellular IAP-1 and -2; NAIP, Neuronal apoptosis inhibitory protein; Ts-IAP, Testis-specific IAP; ILP-2, IAP-like protein 2; TRAF2, Tumor necrosis factor-associated factor 2; α1-ARAs, αl-adrenergic-receptor antagonists; 5-ARIs, 5α-reductase inhibitors; TNFRs, Tumor necrosis factor receptors; Se, Selenium; Ly, Lycopene; SeR, *Serenoa repens*; COX, Cyclooxygenase; LOX, 5-lipoxygenase.
